# Evaluation of the Biodegradability of a Composite Material Reinforced with Cellulose Acetate Nanofibers

**DOI:** 10.3390/polym18141720

**Published:** 2026-07-13

**Authors:** Pedro Rodríguez Sandoval, Andres Felipe Rubiano-Navarrete, Angie Natalia Cuy Talero, Edwin Yesid Gómez-Pachón, Ricardo Vera Graziano

**Affiliations:** 1 Grupo de Investigación de Materiales y Ensayos-GIMES, SENA-Centro de Materiales y Ensayos, Escuela de Posgrado en Ingeniería, Universidad Pedagógica y Tecnológica de Colombia—UPTC, Tunja 150003, Colombia; 2 Grupo de Investigación en Diseño, Innovación y Asistencia Técnica para Materiales Avanzados-DITMAV, Doctorado en Ingeniería y Ciencia de los Materiales, Universidad Pedagógica y Tecnológica de Colombia—UPTC, Tunja 150003, Colombia; 3 Grupo de Investigación en Diseño, Innovación y Asistencia Técnica para Materiales Avanzados-DITMAV, Escuela de Diseño Industrial, Universidad Pedagógica y Tecnológica de Colombia—UPTC, Duitama 150461, Colombia; 4 Instituto de Investigaciones en Materiales, Universidad Nacional Autónoma de México, Coyoacán 04510, Mexico

**Keywords:** composite material, composting, biodegradation

## Abstract

The development of biodegradable composite materials offers a sustainable alternative to conventional synthetic polymers, particularly in short-lived applications. In this context, the incorporation of nanoscale reinforcements can improve mechanical properties without compromising the material’s degradability. The purpose of this study was to evaluate the composting behavior of a composite material based on low-density polyethylene (LDPE), potato starch, and cellulose acetate nanofibers (NFCA). The material’s degradability and mechanical stability were analyzed over 180 days according to the ASTM D5988 standard. The nanofibers were produced via electrospinning, yielding an average diameter of approximately 85 nm. The composite material was prepared using twin-screw extrusion followed by injection molding to produce ASTM D638 test specimens. The effects of biodegradation were evaluated through mass loss, CO_2_ generation in a desiccator, tensile testing, Shore D hardness measurements, and microstructural analysis via scanning electron microscopy (SEM). The results demonstrated a progressive increase in CO_2_ generation, particularly in the reinforced formulations (up to 111.76%). The formulation containing 3% NFCA exhibited the greatest mass loss (20.28%). Tensile testing revealed moderate reductions in maximum stress (1.66–8.44%), whereas hardness increased by up to 5.4% in the reinforced formulations. SEM analysis revealed increased porosity as the composting period progressed. The findings suggest that the incorporation of NFCA enhances biodegradative performance, as evidenced by increased CO_2_ evolution, mass loss, and morphological changes during composting.

## 1. Introduction

In recent decades, growing concern regarding the environmental impact of synthetic polymers has driven a transformation in the development of new materials, particularly those intended for single-use applications and packaging. The increasing demand for packaging materials, particularly in the food, beverage, healthcare, and consumer goods sectors, has significantly increased the consumption of synthetic plastics worldwide. Recent market analyses indicate that the global packaging market exceeded USD 1.1 trillion in 2025, while the plastic packaging sector accounted for more than USD 480 billion and continues to expand due to its low cost, versatility, and processing advantages. Furthermore, the global single-use plastic packaging market is valued at approximately USD 49 billion and is expected to maintain sustained growth in the coming years [1]. These figures highlight the economic importance of plastic-based packaging while underscoring the urgent need to develop sustainable and biodegradable alternatives capable of reducing the environmental impact associated with conventional polymeric materials. In response to these demands, biodegradable composite materials have emerged as viable and sustainable alternatives. These materials combine a polymeric matrix with reinforcements or fillers, where at least one component is of natural and biodegradable origin. This approach seeks to maintain a balance among functionality, strength, and degradability [2]. Traditionally, composite materials have been valued for their enhanced mechanical properties—such as strength, stiffness, and durability—and are widely used in the automotive, aeronautical, and construction sectors. However, current research has shifted toward developing composites that, in addition to exhibiting favorable mechanical properties, undergo controlled degradation under composting conditions, thereby complying with international standards such as ASTM D5988 [3].

Various studies have explored the use of natural reinforcements such as coconut, fique, hemp, cocoa husk, and jute. These reinforcements have been shown to not only improve the physical properties of the composites but also preserve or even enhance their biodegradability. Such materials are attractive due to their availability, low cost, minimal environmental impact, and compatibility with thermoplastic matrices [4].

In this context, nanotechnology has played a fundamental role. The use of nanoscale reinforcements has proven effective in improving the interfacial compatibility between synthetic and biodegradable polymers, as well as in optimizing mechanical properties without compromising the material’s degradability. In particular, cellulose nanofibers (NFCs) have attracted significant interest due to their high specific surface area, low density, tensile strength, and entirely renewable and biodegradable nature. NFCs obtained through methods such as electrospinning have been successfully incorporated into polymeric matrices, generating hybrid materials with superior properties [5].

Recent studies have demonstrated that cellulose nanomaterials can establish strong interfacial interactions with both polymeric matrices and lignocellulosic reinforcements, contributing to improved stress transfer and enhanced composite performance [6]. The effectiveness of these interactions depends on factors such as nanomaterial dispersion, surface chemistry, aspect ratio, and compatibility with the surrounding matrix. In addition, the incorporation of cellulose nanomaterials has been shown to improve stiffness, tensile strength, and structural stability while maintaining the renewable and biodegradable character of the composite [7]. These findings highlight the importance of carefully designing the composition and distribution of reinforcing phases to achieve an optimal balance between mechanical performance and biodegradability.

Electrospinning has been widely utilized to obtain micro- and nanofibers from biopolymers such as cellulose, starch, and proteins. Its versatility in controlling operational variables (e.g., solution viscosity, voltage, flow rate, and injector–collector distance) enables the production of porous structures with high surface-area-to-volume ratios, which are ideal for biodegradable applications [4,8].

Experimentally, several authors have prepared biodegradable composites through extrusion and pelletizing, followed by injection molding to produce “dog-bone” test specimens in accordance with the ASTM D638 standard for tensile testing. These specimens are subsequently subjected to controlled composting conditions (regulating pH, soil moisture, and temperature) under the ASTM D5988 standard to evaluate mass loss, CO_2_ release, and mechanical properties such as maximum stress and hardness [9].

This study evaluates a composite material consisting of a low-density polyethylene (LDPE) matrix, potato starch as the biodegradable component, and electrospun cellulose acetate nanofiber (NFCA) reinforcements. The primary objective is to analyze the material’s behavior and degradability under composting conditions, alongside the effect of the nanoscale reinforcement on its mechanical and morphological properties over a 180-day period, following the guidelines of the ASTM D5988 and ASTM D6400 standards [3,10].

## 2. Materials and Methods

### 2.1. Materials

In accordance with ASTM 5988-03 regarding the preparation of the substrate for degrading polymers, the materials, equipment, and reagents listed in Table 1 were utilized.

### 2.2. Preparation of Raw Materials

#### 2.2.1. Compost Preparation

Activated mature compost was prepared in accordance with the American Society for Testing and Materials (ASTM) D5988-03 standard, maintaining parameters for temperature (24 ± 3 °C), pH (6.0 to 8.0), and moisture (50% to 70%). During soil preparation, moisture levels should remain close to, but never below, 50% to facilitate airflow between soil granules and maintain optimal aerobic conditions. Strict control of soil pH is essential, as the standard cautions: “soil with a pH greater than 8.0 can retain more CO_2_ produced by microorganisms than a neutral soil, and soil with a pH below 6.0 can have an atypical microbial population” [2].

The compost was prepared using 13.5 kg of sorted organic waste, comprising 70% banana peels, 20% cassava peels, and 10% arracacha peels. To accelerate decomposition, this material was reduced to a particle size of 5 mm. The mixture was placed outdoors in 20 L containers, covered with a mesh for overnight storage, and mixed every 4 h during the first 3 days to promote aeration and rapid drying. By the third day, the compost reached maximum organic decomposition, characterized by a moist consistency, brown coloration, and the absence of unpleasant odors.

The material was subsequently spread outdoors to dry, yielding 6.5 kg of dry, mature compost. This was further reduced to a final mass of 6.2 kg to ensure manageability. This final mass was then used to formulate the organic compost mixture in accordance with the ASTM D5988-03 standard, which stipulates that “the test soil may also be a mixture of a natural soil and a mature compost: 1 g of compost per 25 g of soil.”

**Figure 1:**
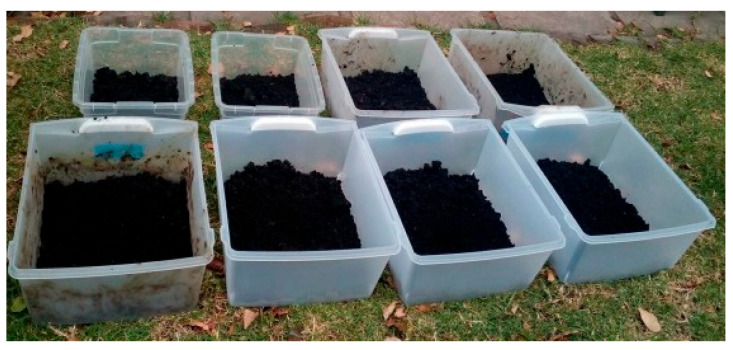
Mixture of organic matter with common soil, arranged in boxes for parameter control.

Figure 1 illustrates the arrangement of the compost in boxes, designed to generate the aerobic conditions required by the manufactured soil.

#### 2.2.2. Raw Material Preparation for Extrusion Process

Initially, potato starch was modified using a mixture of 65% potato starch, 25% polyethylene glycol, and distilled water. The modified potato starch was weighed using a Nimbus digital electronic scale (Adam Equipment Co. Ltd., Milton Keynes, UK) with a capacity ranging from 1 g to 6200 g, while the additives were measured in a 25 mL graduated cylinder according to their respective percentages. These components were then deposited into a 500 mL beaker and mixed manually using a glass stirrer.

Subsequently, the low-density polyethylene (LDPE), potato starch (PS), and cellulose acetate nanofibers (NFCA) were weighed. The LDPE constituted 85% of the mixture’s weight, while the PS accounted for 15%. The NFCA reinforcement was added at percentages of 0.5% and 0.3% of the total mixture weight, resulting in the treatments detailed in Table 2.

### 2.3. Processes

#### 2.3.1. Electrospinning Process

Cellulose acetate nanofibers (NFCA) were produced via electrospinning at the laboratories of the Universidad Pedagógica y Tecnológica de Colombia (UPTC), Duitama campus, and the Materials Research Institute (IIM) of the Universidad Nacional Autónoma de México (UNAM). The process involves introducing a room-temperature solution into a fiber-dispensing tip subjected to a high voltage exceeding 1 kV. The solution protrudes from the needle tip, forming a meniscus that becomes charged due to electrostatic interactions. The resulting electrostatic repulsion, combined with the solution’s inherent surface tension, causes the charged droplet to stretch into a conical geometry.

Under optimal conditions, a critical point is reached wherein the droplet emits a constant flow, yielding a continuous polymer jet [4]. As the jet travels toward the collecting plate, it gradually dries. Initially, the flow remains linear; however, as it moves further from the tip, charge migration generates electrostatic repulsion. This repulsion then induces a whipping instability, ultimately depositing the fiber onto the collecting plate, which serves as the circuit’s ground [11].

#### 2.3.2. Extrusion/Pelletizing Process

The reinforced biodegradable composite material was manufactured in the Thermoplastic Matrix Composite Materials Laboratory at the SENA Centro de Materiales y Ensayos. The process commenced with the setup and programming of the extrusion and pelletizing line according to the treatments listed in Table 3, configuring process variables such as six temperatures, pressures, speeds, screw revolutions per minute (rpm), and cycle times. A 14198 series twin-screw extruder (reference PTL-30 Labtech Engineering, Samut Prakan, Thailand) was utilized, programmed with a heating curve ranging from 50 °C to 165 °C across its five zones, and a screw rotation speed of 40 rpm.

The preheated mixture was introduced into the machine’s feeding hopper and passed through the cylinder. The screws transported the material to the extruder head, which featured a four-strand die. This die generated bioplastic filaments that subsequently passed through a cooling tub, where pressurized water circulated in a closed loop to solidify the material. A pulling roller system then fed the solidified filaments into the pelletizing equipment to produce pellets 4 mm in length. This procedure was repeated for each treatment.

#### 2.3.3. Injection Process

Test specimens for mechanical testing were manufactured in a dog-bone shape according to the ASTM D-638 Type A standard. Injection molding was performed using a two-cavity metallic mold equipped with a cold runner and automatic ejection. A Wittmann Battenfeld injection machine (Wittmann Battenfeld, Kottingbrunn, Austria) with a clamping force of 600 tons and an injection capacity of 150 g, located in the Polymer Transformation Laboratory at the SENA Centro Metalmecánico, was utilized.

After programming the injection parameters according to the treatments outlined in Table 3, the pelletized material was placed in a Shini pre-drying hopper at 80 °C for 15 min to eliminate moisture. The dried pellets were then loaded into the injection machine’s hopper and gravity-fed into the screw chamber. Heating elements positioned along the barrel melted, homogenized, and plasticized the thermoplastic polymer before it was injected into the mold [12].

### 2.4. Preparation for Biodegradation Test

#### 2.4.1. Compost Test Specimen Seeding Process

**Figure 2:**
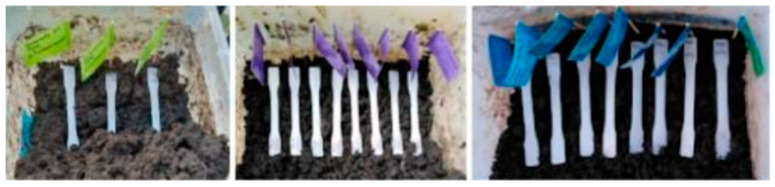
Process of seeding test specimens in the compost.

After the compost control parameters stabilized and the compost matured for 2 weeks, a random sample of six dog-bone test specimens per treatment—manufactured via injection molding according to the ASTM D638 standard—was embedded at a depth of 7 cm, as illustrated in Figure 2. The specimens must be weighed prior to being embedded.

In accordance with the ASTM D5988 standard, these test specimens must remain embedded for a minimum of 180 days. Every 30 days, a random batch of specimens from each treatment must be extracted and weighed to measure the mass loss resulting from the material’s degradation processes.

#### 2.4.2. Chemical Analysis of CO_2_ Collection Using a Desiccator

**Figure 3:**
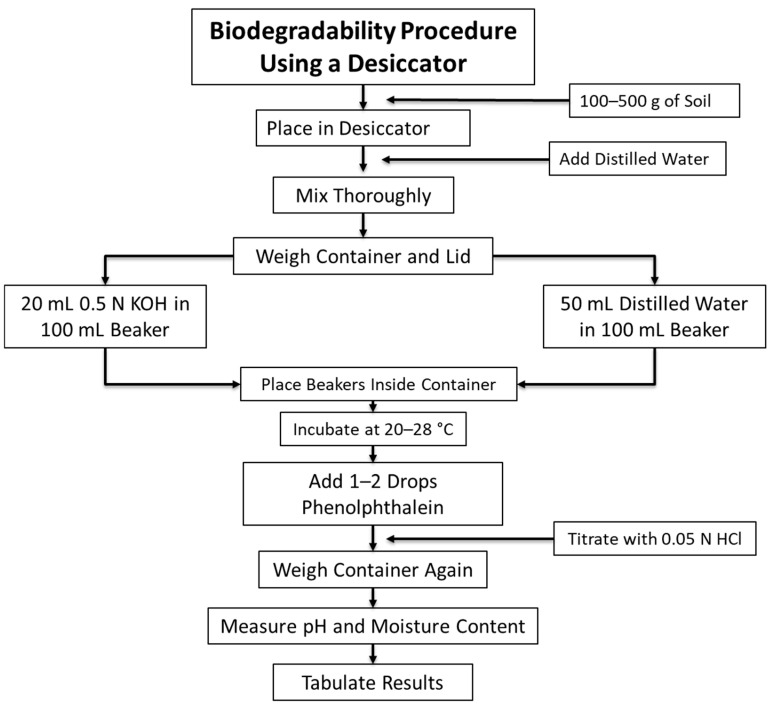
Biodegradation procedure using a desiccator.

A sample of the compost containing the buried test specimens was collected to determine the amount of carbon dioxide released. This analysis followed Procedure 7 of the ASTM 5988-03 standard, which involves placing the soil and a single test specimen into a desiccator. A solution comprising 20 mL of 0.5 N potassium hydroxide and 50 mL of distilled water was utilized as a carbon dioxide trap. This procedure is illustrated in Figure 3.

After the sample is mixed in the desiccator, it must incubate for 7 days. Subsequently, the KOH trap is removed and titrated with 0.25 N hydrochloric acid via the volumetric method, as shown in Figure 3. This process determines the amount of carbon dioxide produced, as it is directly proportional to the volume of acid consumed [13,14].

### 2.5. Mechanical Testing of Specimens During the Biodegradation Process

#### 2.5.1. Tensile Testing

This test evaluates the characteristics of a reinforced polymeric material subjected to tensile stress during the biodegradation process. Tensile tests were conducted using a Universal testing machine (BESMAK, Ankara, Türkiye) equipped with a 5-ton load cell, located in the polymer mechanical testing laboratories of the SENA Centro de Materiales y Ensayos (Regional Distrito Capital). Dog-bone specimens, obtained via injection molding for the different bioplastic treatments (T1, T2, and T3), were tested in accordance with the ASTM D638 standard. The specimens were secured in the machine’s grips, and a test speed of 5 mm/s was applied until rupture. The machine’s software facilitates the generation of a test graph and allows the results to be saved for subsequent analysis.

#### 2.5.2. Hardness

Hardness is defined as a material’s resistance to penetration or scratching under an applied force. Testing was performed using a Check-Line Digital durometer (Model MSDD 4AD00, Bareiss Prüfgerätebau GmbH, Oberdischingen, Germany) equipped with a Shore D hardened steel indenter. For this test, a random sample of five specimens from each studied treatment was used. Each specimen was positioned under the indenter, which applied a penetration force to leave an impression on the material. Hardness was then measured in accordance with the ASTM D220 standard.

#### 2.5.3. Mass

The mass of the specimens used in the biodegradation test for the studied treatments was verified using a Nimbus digital electronic scale (Adam Equipment Co. Ltd., Milton Keynes, UK) with a capacity of 1 g to 6200 g and a readability of 0.1 g. These data were recorded monthly for analysis over the six-month testing period.

### 2.6. Physical Characterization of Specimens During the Biodegradation Process

Structural analysis of the reinforced composite material during biodegradation was performed on the fractured specimen fragments remaining after tensile testing.

#### Scanning Electron Microscopy (SEM)

Scanning electron microscopy (SEM) was utilized to observe and compare the microstructural evolution of the treatments during biodegradation. Analyses were performed using a Phenom XL scanning electron microscope (Thermo Fisher Scientific, Eindhoven, The Netherlands). Cross-sectional samples from the rupture areas of specimens from treatments T1, T2, and T3 were mounted on SEM pin stubs and secured with copper tape. To improve electrical conductivity and image quality, the samples were coated with a thin layer of gold; no contrast agent was employed. Micrographs were subsequently acquired using an accelerating voltage of 20 kV.

## 3. Results

ASTM 638 [15] dog-bone test specimens were produced in order to conduct the biodegradation testing of the reinforced composite material. The resulting parameters are listed below:

### 3.1. Processes

#### 3.1.1. Electrospinning Process

**Figure 4:**
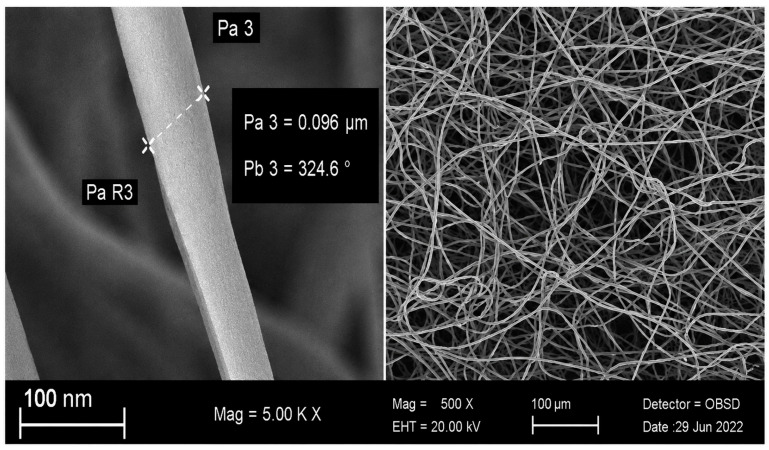
Fiber diameter and membrane structure results.

Electrospinning was conducted at the laboratories of the UPTC and the UNAM. After evaluating various solution types and modifying the electrospinning parameters across more than 100 treatments, nanofibers with an average diameter of 85 nm and a uniform, bead-free structure without ruptures were successfully produced (see Figure 4). The process setup was standardized using the values presented in Table 3.

The preparation of these nanofiber membranes was guided by the research of Ochica, Muñoz, Gómez, Maciel, and Rivera [16].

#### 3.1.2. Extrusion/Pelletizing Process

To adjust the extrusion and pelletizing parameters for this biodegradable composite material, prior research on bioplastic extrusion and polymer reinforcement by Rodriguez, P., Prieto, E., Pachón. [8] was analyzed.

Treatments were programmed according to the experimental design outlined in Table 1. The configuration that yielded process stability and a high-quality product was standardized; the resulting variable values are presented in Table 4.

**Figure 5:**
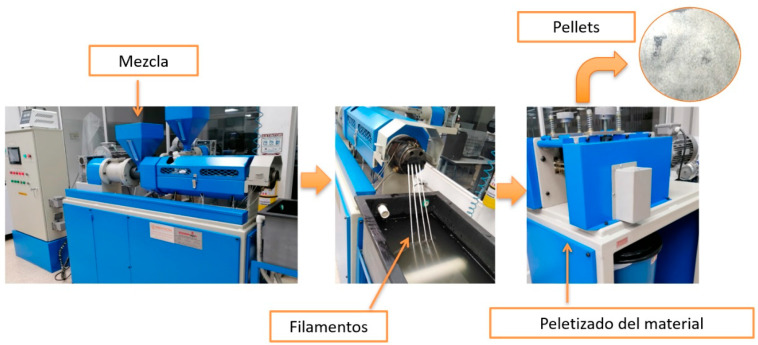
Extrusion/pelletizing process of the reinforced biodegradable material.

As the extruded composite material passed through the die head, filaments with a diameter of 4 mm were produced. These filaments were routed through a 10 °C water cooling tank and subsequently conveyed via a roller puller to the pelletizer, yielding pellets 4 mm in diameter and 4 mm in length. For each treatment listed in Table 1, 500 g of material were collected to prepare test specimens for physical property characterization (see Figure 5).

#### 3.1.3. Test Specimen Injection Process

**Figure 6:**
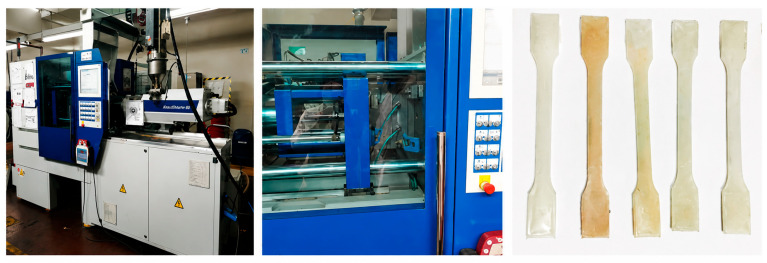
Injection process of ASTM D 638 test specimens.

Using the injection molding process, 20 injections were performed for the treatments listed in Table 2. The average weight per injection was 26 g, yielding a total of 80 ASTM D-638 test specimens intended for mechanical property characterization and biodegradation testing. To inject the composite material—composed of potato starch and low-density polyethylene, and reinforced with cellulose acetate nanofibers—the machine was configured using the experimental design variables detailed in the master’s thesis and research of Rodriguez, Prieto, and Pachón [8] (see Figure 6).

### 3.2. Biodegradability Test of the Reinforced Composite Material

#### 3.2.1. Composting

The stabilization process for carbon dioxide production was controlled by regulating pH, moisture, and temperature. The mature compost was distributed across eight boxes to establish the aerobic conditions required by the manufactured soil. The compost was successfully stabilized by maintaining the parameters specified by the American Society for Testing and Materials (ASTM) Standard D5988-03: a temperature of 24 ± 3 °C, a pH between 6.0 and 8.0, and a moisture level between 50% and 70%.

Data in Table 5 concerning pH, temperature, and soil moisture indicate that the initial soil moisture remained below the levels specified by the ASTM 5988-03 standard for degrading polymeric materials. Therefore, after the test specimens—containing varying proportions of potato starch in the synthetic matrix—are embedded, the moisture must be increased to 50%. Through constant aeration, the moisture is subsequently raised to 70% to activate the soil and facilitate the degradation of the material under study.

Once the soil was prepared, the ASTM D-638 test specimens from treatments T1 (85% low-density polyethylene, 15% potato starch, 0% NFCA), T2 (84.5% low-density polyethylene, 15% potato starch, 0.5% NFCA), and T3 (82% low-density polyethylene, 15% potato starch, 3% NFCA) were embedded to ensure decomposition. This activation of the soil, achieved by increasing the moisture to 70%, enables the biodegradation process to occur over a 6-month period [4].

#### 3.2.2. Measurement of CO_2_ Generation in the Desiccator

**Figure 7:**
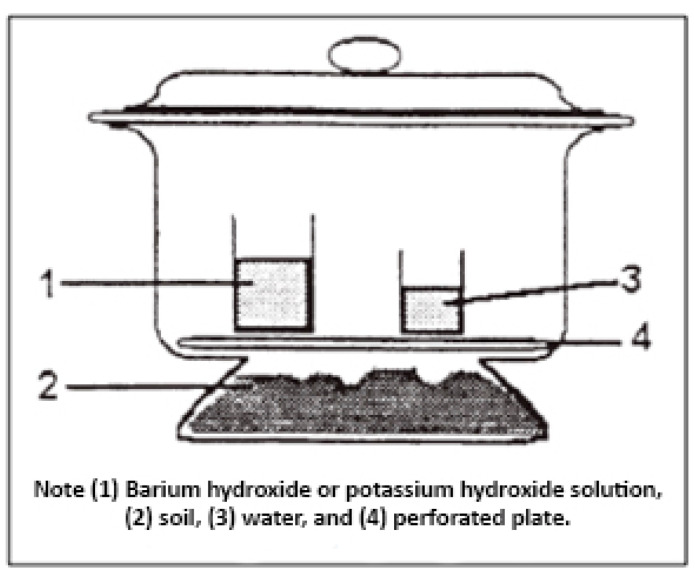
Analysis of carbon dioxide (CO_2_) in the desiccator.

**Figure 8:**
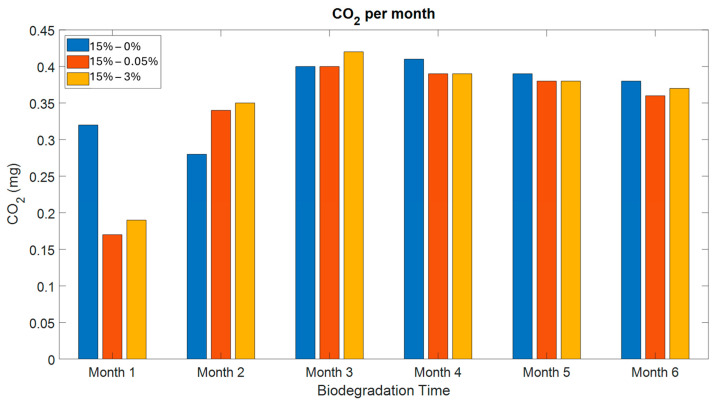
CO_2_ analysis results over 6 months.

Carbon dioxide (CO_2_) analysis was performed in a desiccator according to Item 11.7 of the ASTM D5988 standard, as illustrated in Figure 7 and Figure 8. Compost soil samples were sieved (mesh size < 2 mm). A 300 g compost sample was weighed, transferred to the desiccator, and mixed with 30 mL of an ammonium phosphate solution. This ammonium phosphate solution was prepared by diluting 4.72 g of the solute to a volume of 100 mL. A barium hydroxide [Ba(OH)_2_] solution was prepared by diluting 0.39 g of Ba(OH)_2_ to a volume of 100 mL. Next, 100 mL of the Ba(OH)_2_ solution was added to a beaker, along with 50 mL of distilled water [17].

In a separate beaker, the surface layer of the Ba(OH)_2_ solution was gently agitated to ensure continuous CO_2_ absorption. The Ba(OH)_2_ solution was subsequently removed from the desiccator, transferred entirely to an Erlenmeyer flask, and one drop of phenolphthalein was added [17]. The desiccator was left uncovered for 30 min, after which a fresh Ba(OH)_2_ solution was introduced. Separately, 0.42 g of hydrochloric acid (HCl) was weighed and diluted to a volume of 100 mL. The Ba(OH)_2_ and phenolphthalein mixture was then titrated. The results are presented in Figure 8.

**Figure 9:**
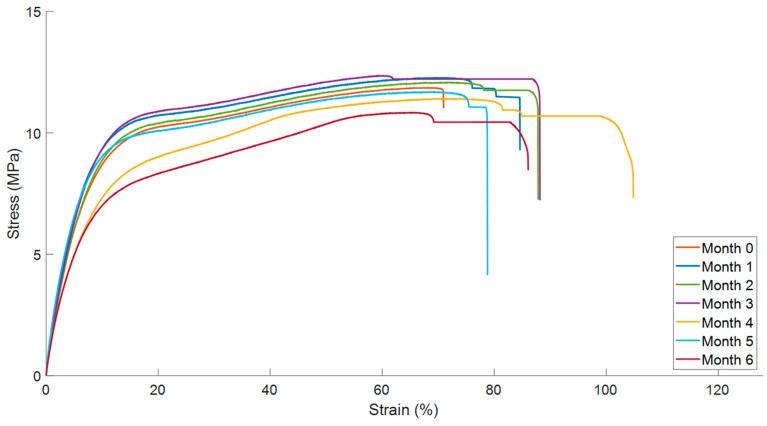
Treatment T2 (LDPE 84.5–Starch 15–NFCA 0.5%) curves per month of the biodegradability test.

Figure 9 presents the average monthly CO_2_ extraction from the compost sample analyzed in the desiccator. In the graph, the bars represent the treatments by color: blue for T1, orange for T2, and gray for T3. The *x*-axis indicates the trial months, and the *y*-axis shows the milligrams of CO_2_.

Following the parameters of the ASTM D-5988 standard for CO_2_ production in the desiccator (Figure 7), carbon dioxide levels increased from the first to the sixth month. For the composite material (potato starch reinforced with cellulose acetate nanofibers), the average CO_2_ increases were 20.93% for T1, 111.76% for T2, and 92.10% for T3. Compared to the non-reinforced starch reference treatment, the reinforced treatments exhibited an 84% greater increase in CO_2_ production. This method confirmed faster degradation in nanofiber-reinforced treatments.

#### 3.2.3. Mechanical Tests

##### Tensile Tests on Biodegradation Specimens

**Figure 10:**
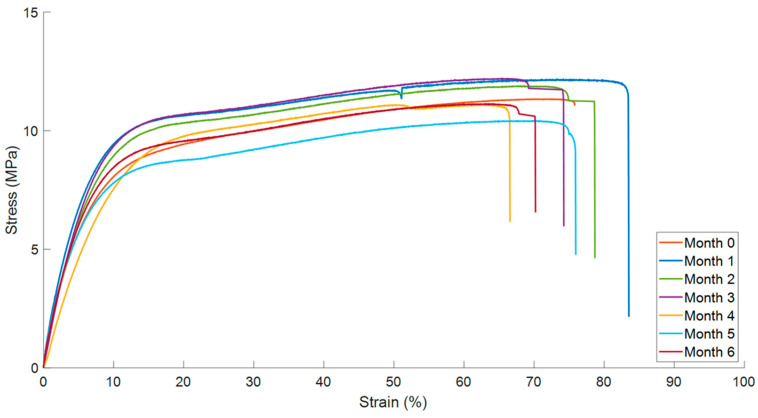
Treatment T3 (LDPE 82–Starch 15–NFCA 3%) curves per month of the biodegradability test.

For the tensile tests, three specimens (*n* = 3) were randomly selected from each treatment at each evaluation period. The stress–strain curves presented in Figure 9 and Figure 10 correspond to the average response of the three replicates. Specimens from treatments T2 and T3 were tested before composting (Month 0) and monthly throughout the 180-day biodegradation period. These results illustrate the evolution of the mechanical behavior of the composite during composting.

The stress–strain curves indicate a progressive reduction in tensile strength and elongation after Month 3 for both treatments compared with the unaged specimens. According to the ASTM D6400 criteria, these changes are consistent with biodegradation-related behavior under controlled composting conditions. The progressive decrease in mechanical performance reflects the structural modifications occurring within the composite as biodegradation advances [18,19].

Figure 10 presents the average stress–strain curves obtained from three replicates (*n* = 3) for treatment T2. The control specimens (Month 0) exhibited an average maximum tensile strength of 11.822 MPa, whereas the specimens composted for 180 days reached an average value of 10.824 MPa, corresponding to an 8.44% reduction. This gradual decrease in tensile strength indicates that biodegradation affected the mechanical performance of the composite while preserving a significant portion of its load-bearing capacity throughout the evaluation period.

Figure 10 displays the results for treatment T3. The black curve again represents the initial control specimen, with a maximum recorded stress of 11.325 MPa. The purple curve illustrates the behavior of the specimen composted for 6 months, which reached a maximum stress of 11.137 MPa. Here, the decrease was only 1.66%, indicating greater mechanical stability compared to the previous treatment.

The more significant loss of strength observed in Figure 10 suggests that the T2 composite formulation is more susceptible to degradation, possibly due to a higher proportion of biodegradable components or lower compatibility with the LDPE matrix. Conversely, treatment T3 exhibits minimal degradation, suggesting that its composition and structure provide better resistance to biodegradation or superior mechanical stability under these test conditions.

Table 6 summarizes the maximum tensile strength values obtained for treatments T2 and T3 during the 180-day composting period, expressed as the mean ± standard deviation (*n* = 3). The results confirm the trends observed in the stress–strain curves, showing an initial increase in tensile strength during the first three months, followed by a progressive reduction as biodegradation advanced. Treatment T2 exhibited a more pronounced decrease in tensile strength, declining from 11.82 ± 0.07 MPa to 10.82 ± 0.14 MPa after 180 days, whereas treatment T3 showed only a slight reduction, from 11.33 ± 0.04 MPa to 11.14 ± 0.12 MPa. The relatively low standard deviation values indicate good repeatability of the tensile measurements among the three replicates, supporting the reliability of the reported mechanical behavior under controlled composting conditions.

##### Hardness Tests on Biodegradation Specimens

**Figure 11:**
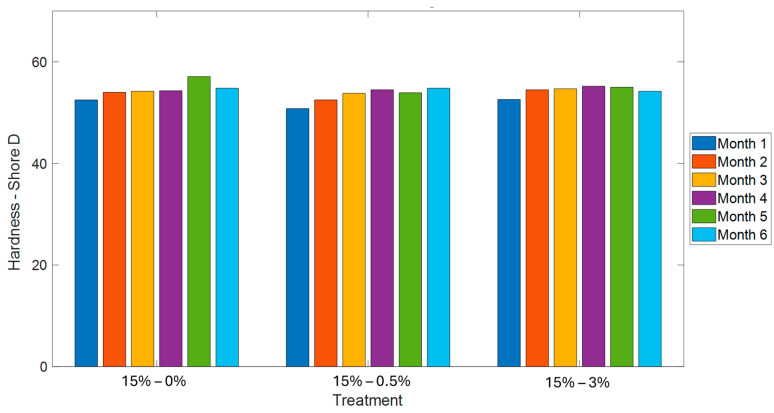
Hardness of treatments during the biodegradation process.

For this test, 5% of the specimens undergoing biodegradation across the three treatments were selected during the 6-month controlled study; the results are shown in Figure 11.

The hardness test results demonstrate the average monthly value for each treatment during the biodegradation process. In the unreinforced treatment T1 (85% LDPE, 15% potato starch, 0% NFCA), the hardness value increased by 4.08% from Month 1 to Month 6. In the reinforced treatments T2 (84.5% LDPE, 15% potato starch, 0.5% NFCA) and T3 (82% LDPE, 15% potato starch, 3% NFCA), hardness increased by an average of 5.4%. The difference in hardness increase between the unreinforced and reinforced treatments was an average of 32.42% [15].

##### Mass Verification of Specimens During the Degradation Process

**Figure 12:**
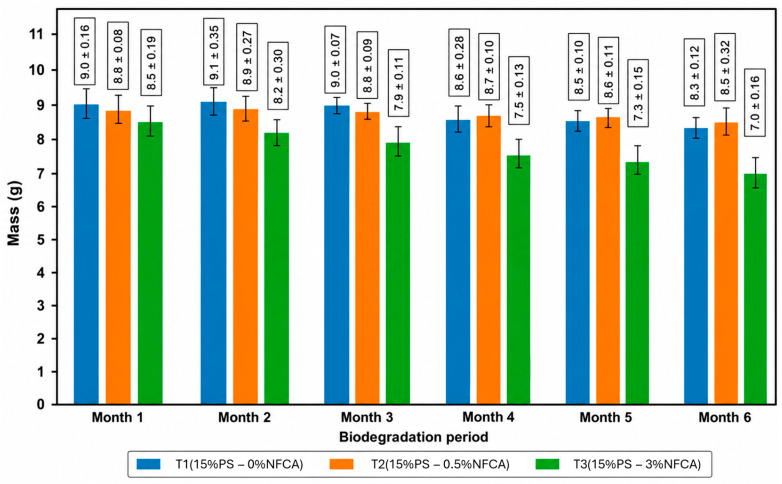
Average mass of specimens during the biodegradation process (mean ± standard deviation, *n* = 3).

Specimens recovered from treatments T1, T2, and T3 were weighed monthly throughout the 180-day composting period. For each treatment and evaluation time, three specimens (*n* = 3) were analyzed, and the results are presented as the mean ± standard deviation (Figure 12).

Figure 12 shows the progressive reduction in specimen mass during composting for all evaluated treatments. Treatment T3 (82 wt.% LDPE, 15 wt.% potato starch, and 3 wt.% NFCA) exhibited the greatest mass loss, with the average mass decreasing from 8.50 ± 0.19 g in Month 1 to 7.00 ± 0.16 g after 180 days. Treatment T2 showed a smaller reduction, from 8.82 ± 0.08 g to 8.50 ± 0.32 g, whereas treatment T1 presented the lowest variation, decreasing from 9.00 ± 0.16 g to 8.30 ± 0.12 g over the same period.

The relatively low standard deviation values obtained for all treatments indicate good repeatability of the measurements among the three evaluated specimens, supporting the reliability of the reported mass-loss results. The greater reduction in mass observed for treatment T3 is attributed to its higher content of biodegradable components, particularly potato starch and cellulose acetate nanofibers, which increase the hydrophilic character of the composite. Greater water absorption facilitates the penetration of moisture and microorganisms into the material, promoting enzymatic fragmentation, degradation of the biodegradable phases, and subsequent mineralization under composting conditions [2,13]. These observations are consistent with the CO_2_ evolution and SEM analyses, both of which indicate a progressive increase in biodegradation throughout the evaluation period.

### 3.3. Scanning Electron Microscopy (SEM)

**Figure 13:**
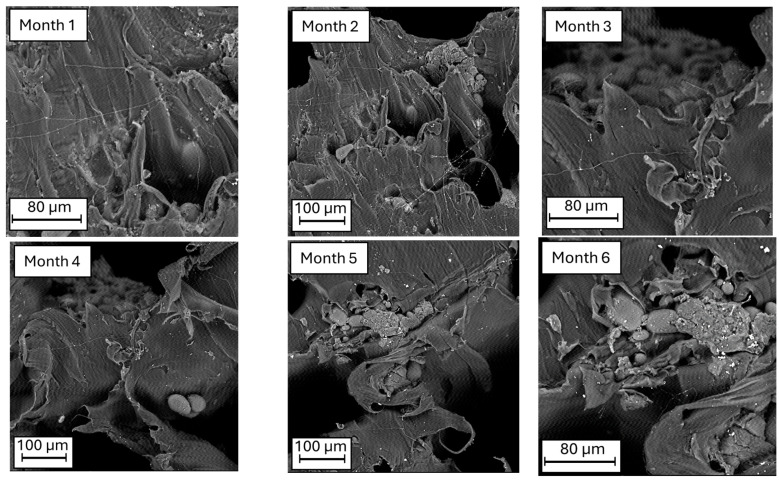
SEM images of treatment T3 for each month of the biodegradation process.

This analysis was performed using the fractured portions of the tensile test specimens from the evaluated treatments. The fracture surfaces were examined by scanning electron microscopy (SEM) to assess the microstructural evolution of the composite during composting. Figure 13 presents the SEM micrographs of treatment T3 collected monthly throughout the 180-day degradation period.

In these images, white circles indicate the presence of cellulose acetate nanofibers (NFCAs), whereas orange circles highlight pores and voids formed during the biodegradation process. During the initial stage (Month 1), the fracture surface exhibited a relatively compact and homogeneous morphology, with a limited number of discontinuities and isolated pores. As the composting period progressed, an increase in the number, size, and distribution of pores and voids was observed, indicating progressive microstructural deterioration.

By Months 3 and 4, larger cavities and a more irregular surface morphology became evident, suggesting the preferential degradation of the biodegradable phases within the composite. At the end of the evaluation period (Month 6), the material exhibited a markedly heterogeneous structure characterized by a higher density of pores and interconnected voids distributed throughout the fracture surface. These features are consistent with the mass loss and CO_2_ evolution results obtained during composting.

Despite the increase in porosity, cellulose acetate nanofibers remained visible throughout the six-month evaluation period, indicating their persistence within the polymeric matrix. The progressive formation of pores and voids, together with the retention of fibrillar structures, suggests that biodegradation primarily affected the more susceptible components of the composite while preserving a significant portion of the load-bearing structure. This observation is consistent with the relatively limited reduction in tensile strength recorded for treatment T3 and supports the progressive nature of the degradation process under controlled composting conditions.

**Figure 14:**
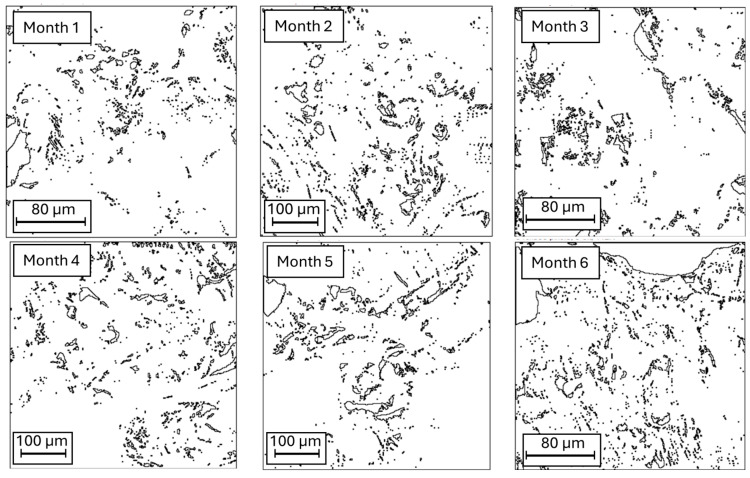
Binary images obtained from ImageJ software for treatment T3 during the composting period, used for apparent porosity quantification through area fraction analysis.

To complement the qualitative SEM observations, the apparent porosity of treatment T3 was quantified using ImageJ software version 1.53 through image thresholding and area fraction analysis. Figure 14 presents the processed images obtained from ImageJ after binary segmentation, which were used to identify and quantify the porous regions developed during the biodegradation process. The results revealed a progressive increase in porosity throughout the composting period, rising from 3.5% in Month 1 to 18.4% in Month 6. Intermediate values of 5.1%, 7.8%, 10.2%, and 13.6% were obtained for Months 2, 3, 4, and 5, respectively.

Overall, the apparent porosity increased from 3.5% in Month 1 to 18.4% in Month 6, evidencing a progressive evolution of the material’s internal structure during the composting process. This increase in porous area quantitatively confirms the microstructural deterioration observed in the SEM micrographs and indicates the gradual removal of biodegradable constituents from the composite. The formation of pores and voids is consistent with the mass loss and CO_2_ evolution recorded during composting, suggesting that biodegradation promoted the development of discontinuities within the material while a significant portion of the load-bearing polymer matrix remained structurally intact.

## 4. Discussion

The results demonstrate a characteristic behavior of hybrid LDPE/TPS systems reinforced with cellulosic structures, where biodegradation is primarily governed by phase interactions and the accessibility of microorganisms to the matrix. The progressive increase in CO_2_ generation and the mass loss observed in the evaluated treatments confirm the occurrence of active biodegradation. This behavior is consistent with findings for LDPE/TPS blends under controlled composting conditions, where the degradation of the hydrophilic phase induces the formation of pores and channels that facilitate material fragmentation [12]. This mechanism implies that the TPS phase acts as a degradation initiator, generating a network of discontinuities that progressively exposes the LDPE matrix to microbial agents.

The observed mass loss values (up to 20.28%) are comparable to those reported for LDPE biocomposites reinforced with lignocellulosic fillers, which exhibit losses between 13% and 31% depending on environmental conditions [20]. This wide range reflects the process’s sensitivity to variables such as temperature, relative moisture, and microbial diversity, which influence the degradation kinetics of the natural phase.

In this study, the selective degradation of TPS increased the material’s surface and internal porosity, facilitating the subsequent degradation of the polymeric matrix. This was confirmed by microstructural analysis, which revealed the formation of cavities and the loss of interfacial cohesion characteristic of these systems.

However, nanoscale cellulosic reinforcements exhibit dual behavior depending on their morphology, degree of crystallinity, and aspect ratio. In LDPE/TPS systems reinforced with cellulose nanocrystals (CNCs), previous studies report significant improvements in mechanical properties and interfacial interaction, but a decreased biodegradation rate due to the increased tortuosity of the polymeric network [21].

This contrasts with the present results, in which the incorporation of cellulose acetate nanofibers (NFCAs) promoted biodegradation. This difference can be explained by the morphological characteristics of the reinforcement: while CNCs have high crystallinity and act as a physical barrier to water diffusion and microbial penetration, NFCAs have lower relative crystallinity and a greater accessible specific surface area. Consequently, NFCAs can create preferential pathways for water diffusion and microbial colonization without forming a similarly tortuous barrier.

This behavior aligns with studies on cellulose-reinforced polylactic acid biocomposites, which demonstrate that microcellulose accelerates biodegradation by acting as a hydrophilic channel, whereas CNCs can retard it; however, combining the two generates synergistic effects [22]. In this context, the NFCAs in the current study appear to play a role analogous to cellulosic structures with lower crystalline ordering, promoting degradation without completely compromising the composite’s structural integrity.

Regarding interfacial compatibility, the mechanical parameters—moderate decreases in tensile strength and an increase in hardness—suggest that functional stress transfer is maintained within the material during the initial stages of biodegradation. This behavior is consistent with studies in which the chemical modification of cellulosic fibers improved interactions with polyolefin matrices, thereby increasing mechanical strength and reducing water absorption [23]. In the present system, TPS as an intermediate phase may act as a natural compatibilizer between the LDPE and NFCAs, reducing the interfacial energy between these physicochemically contrasting components.

This hypothesis is supported by reports on LDPE/cellulose acetate systems, which exhibit marked phase immiscibility that results in heterogeneous structures if compatibilizing agents are omitted [24]. Consequently, the inclusion of TPS in the studied system not only contributes to biodegradability but may also improve interfacial cohesion between components, acting as a compatibility bridge that reduces stress concentration at the interface.

Finally, the increase in hardness observed after the biodegradation tests can be attributed to multiple concurrent factors. First, the selective degradation of the amorphous starch phase leads to a relative increase in the crystalline fraction of the LDPE, thereby increasing the material’s rigidity. Additionally, the loss of TPS—which acts as an internal plasticizer for the polymeric network—can induce the post-crystallization of previously restricted LDPE segments. This phenomenon is frequently reported in polymeric systems subjected to hydrolytic or microbial degradation, where the removal of the soft phase favors the molecular reorganization of the matrix.

Overall, the results indicate that biodegradation in LDPE/TPS systems reinforced with cellulosic structures is a multifactorial process dependent on reinforcement morphology, interfacial compatibility, and environmental conditions. Specifically, the incorporation of NFCAs facilitates an appropriate balance between mechanical properties and degradability. This distinguishes these composites from systems based exclusively on CNCs, where biodegradation can be limited by the barrier effect of the crystalline network [21,22].

## 5. Conclusions

The results obtained from the biodegradation tests of the reinforced composite material were as follows:

1. Initially, cellulose acetate nanofibers were fabricated via electrospinning, yielding filaments with a homogeneous structure and diameters between 86 and 100 nm. Standardizing this process enabled continuous production, facilitating its integration into the synthetic polymer and starch blend according to the percentages established for the experimental treatments. Subsequently, dispersive mixing of the three components (LDPE, potato starch, and cellulose nanofibers) and the required additives was performed using twin-screw extrusion to produce composite pellets. Finally, for each evaluated treatment, these pellets were injection-molded into dumbbell-shaped specimens in accordance with the ASTM D638 standard.
2. Compost preparation followed the ASTM D5988-03 methodology; controlling key variables such as pH, temperature, and soil moisture yielded a total of 13.5 kg of compost. The biodegradation process was initiated by burying the specimens in compost boxes at a depth of 7 cm, spaced 10 cm apart, according to their respective treatments. Samples were extracted monthly over the 6-month study period. To measure the generated CO_2_, 200 g of compost per treatment were analyzed using a desiccator system. The results indicated a progressive increase in CO_2_ generation across all three treatments. This increase was more pronounced in the reinforced treatments (T2 and T3), which recorded an 86% increase compared to Treatment T1. These results confirm that the three materials underwent active biodegradation, fulfilling the criteria established by the ASTM D5988-03 standard.
3. During tensile mechanical testing, the results expressed as the mean ± standard deviation (*n* = 3) showed good repeatability among replicates. Treatment T2 (LDPE 82%, PS 15%, NFCA 0.5%) exhibited a progressive decrease in maximum stress starting in Month 3. By the end of the 6-month composting period, this reduction reached 8.66%, yielding a final value of 10.82 MPa. Conversely, Treatment T3 (LDPE 82%, PS 15%, NFCA 3%) demonstrated a lower loss of strength, recording a 1.88% decrease over the same period. Overall, maximum stress exhibited an average reduction of 5.27%, indicating that cellulose acetate nanofibers contributed to preserving the mechanical integrity of the composite during biodegradation by improving the cohesion between the polymeric matrix and the starch, as supported by scanning electron microscopy (SEM) observations.
4. Regarding hardness, a progressive increase was observed over the composting period. In the unreinforced treatment (T1), the average increase was 4.08%, whereas the reinforced treatments (T2 and T3) recorded an increase of 5.4%. This is attributed to surface hardening caused by the loss of biodegradable phases.
5. Regarding mass loss, all three treatments exhibited a continuous monthly reduction during biodegradation. Treatment T3 (LDPE 82%, PS 15%, NFCA 3%) presented the greatest mass loss, reaching an accumulated value of 20.28% after 180 days. This indicates greater susceptibility to degradation due to its higher cellulose nanofiber and starch content.
6. SEM imaging revealed that as degradation progressed, the initially homogeneous material structure underwent notable changes over the months, including the appearance of voids and pores. Nevertheless, NFCAs remained visible within the material’s microstructures at each analysis stage, confirming their effective integration into the polymeric matrix.
7. Porosity analysis using ImageJ revealed a progressive increase in the apparent porous area of treatment T3 throughout the 180-day composting period, from 3.5% to 18.4%. This increase confirms the development of microstructural discontinuities associated with biodegradation and provides additional evidence of the degradation process observed by SEM. Furthermore, the evolution of porosity showed good agreement with the mass loss and CO_2_ generation results, demonstrating the usefulness of image analysis as a complementary tool for evaluating the biodegradation behavior of polymeric composites.
8. In accordance with ASTM D6400 standard criteria, the evaluated treatments exhibited biodegradative performance under controlled composting conditions, as evidenced by CO_2_ evolution, mass loss, changes in mechanical properties, and SEM observations.
9. In this regard, the developed composite material, comprising a thermoplastic LDPE matrix reinforced with potato starch and NFCAs, demonstrates potential for applications where a balance between mechanical performance and biodegradability is desirable. The results obtained under controlled composting conditions indicate that the material is capable of undergoing biodegradation-related changes while retaining a significant portion of its mechanical integrity during the evaluation period. These findings provide a basis for future studies aimed at assessing its suitability for specific industrial applications, particularly those requiring a short service life and improved end-of-life management.

## Figures and Tables

**Table 1:** Materials, equipment and reagents.

Materials	Equipment	Reagents
Black soil, fertile soil Organic waste (banana, cassava, and carrot peels). Beakers; perforated ceramic plates or other inert support. Burette.	Analytical equipment (Thermo Fisher Scientific, Waltham, MA, USA) for measuring total carbon content in the test sample Analytical balance (Sartorius Entris II BCE224I-1S, Sartorius AG, Göttingen, Germany). Oven OMH400 (Thermo Fisher Scientific, Waltham, MA, USA) for moisture determinations. Muffle furnace (Thermolyne F6010, Thermo Scientific, Dubuque, IA, USA), set at 550 °C for ash determination. pH meter (Seven2Go™ S2, Mettler Toledo, Greifensee, Switzerland). Darkened chambers or cabinets maintained at room temperature.	Ammonium phosphate, 4.72 g/L. Potassium hydroxide 0.5 N. Hydrochloric acid, 0.25 N.

The biodegradable composite material was prepared using low-density polyethylene (LDPE; POLIFÉN 641, Ecopetrol, Bogotá, Colombia), modified potato starch (Fécula de papa, Emsland Group, Emlichheim, Germany), polyethylene glycol (PEG; Ref. 6000, Batch 200115W001203, Chemicol CH S.A.S., Bogotá, Colombia), and distilled water (Ref. destilada, CEPROSA, Bogotá, Colombia). Cellulose acetate nanofibers (NFCA) were manufactured using cellulose acetate (Ref. 419028, Sigma-Aldrich, St. Louis, MO, USA), acetone (Ref. 179973-4L, Sigma-Aldrich), ethanol (Ref. 100983–100, Emsure, Darmstadt, Germany), and chloroform (Ref. 918003-4L, J.T. Baker, Phillipsburg, NJ, USA).

**Table 2:** Experimental Design Treatments.

Treatments	Blend Composition (%)		
T	LDPE	PS	NFCA
T1	85	15	0
T2	84.5	15	0.5
T3	82	15	3.0

These materials were integrated using a Shini vertical industrial mixing hopper at a speed of 120 rpm for 10 min. The mixture was subsequently packaged in labeled bags according to the experimental design treatments [4].

**Table 3:** Electrospinning process variables.

Item	Variable	Parameters	Units
1.	Flow	30	mL/h
2.	Voltage	17	kV
3.	Distance	30	cm
4.	Time	9.50	minutes
5.	Volume	5	mL
6.	Concentration	6	%

**Table 4:** Extrusion/pelletizing process parameters.

Process Parameters: Extrusion/Pelletizing	
Temperatures (°C)	Zone 1: 50
	Zone 2: 80
	Zone 3: 100
	Zone 4: 140
	Zone 5: 165
Cooling tank temperature (°C)	10
Extruder screw speed (RPM)	30
Pelletizer speed (RPM)	40
Extruder motor frequency inverter (Hz)	50
Pelletizer motor frequency inverter (Hz)	60

**Table 5:** Control of the pH, Temperature, and Soil Moisture variables of the manufactured soil.

Box with Manufactured Soil	pH	Temperature (°C)	Soil Moisture (%)	Relative Soil Moisture (%)
C1	7.224	21	43.908	47
C2	7.744	18	41.309	47
C3	7.026	17	39.900	47
C4	7.313	18	43.463	47
C5	7.936	20	40.350	47
C6	7.329	18	43.383	47
C7	7.627	18	41.894	47
C8	7.489	18	42.548	47

**Table 6:** Maximum tensile strength of treatments T2 and T3 during the biodegradation process. Results are expressed as the mean ± standard deviation (*n* = 3).

Composting Time (Months)	Treatment T2 (15% PS—0.5% CANF)	Treatment T3 (15% PS—3% CANF)
	Mean ± SD	Mean ± SD
0	11.82 ± 0.7	11.33 ± 0.4
1	12.01 ± 0.07	12.01 ± 0.09
2	12.18 ± 0.16	12.10 ± 0.37
3	12.25 ± 0.6	12.16 ± 0.86
4	11.65 ± 0.9	11.60 ± 0.11
5	11.24 ± 0.13	11.16 ± 0.12
6	10.82 ± 0.14	11.14 ± 0.12

## Data Availability

The original contributions presented in the study are included in the article, further inquiries can be directed to the corresponding author.
